# Antibody induced by one-dose varicella vaccine soon became weak in children: evidence from a cross-sectional seroepidemiological survey in Beijing, PRC

**DOI:** 10.1186/s12879-015-1236-x

**Published:** 2015-11-10

**Authors:** Luodan Suo, Li Lu, Meng Chen, Xinghuo Pang

**Affiliations:** Beijing Center for Disease Control and Prevention (CDC), Capital Medical Unversity School of Public Health and Family Medicine, 16 Hepingli Zhongjie, Dongcheng Dist, Beijing 100013 P. R. China

**Keywords:** Chickenpox, Varicella, Seroprevalence, Vaccine failure

## Abstract

**Background:**

Numerous post-licensure studies, mostly from field epidemiological evidences such as outbreak surveys, have demonstrated the effectivenesss and insufficiency of one-dose varicella vaccine in outbreak control. Serological evidence of immunization failure is, however, relatively less reported in contrast. A cross-sectional seroepidemiological survey of Beijing residents was performed in 2012 in the People’s Republic of China, after the one-dose varicella vaccine had been widely used for several years.

**Methods:**

Multistage stratified random sampling method was designed to recruit 2 144 subjects. The ELISA method was used to test the present blood samples collected and the reserve samples collected in 2008 to assess the trends of anti-VZV seroprevalence in the past 5 years and to determine the risk factors for varicella infection.

**Results:**

The age- and sex- adjusted overall anti-VZV seropositivity of Beijing residents in 2012 was 84.5 %. Two groups’ adjusted overall anti-VZV seroprevalence in 2012 showed obvious growth compared with 2008 (<1 yr old: from 6.3 % to 16.9 %; 1-4 yr old: from 27.6 % to 57.2 %). Reported one-dose vaccination history was 71.6 % (149/208), 80.9 % (182/225) and 82.2 % (180/219) in the 1-4 yr, 5-9 yr, 10-14 yr age groups, respectively. Of subjects who had received the one-dose vaccine, 36 % (216/603) showed negative anti-VZV concentrations (<110 mIU/mL); additionally 15.9 % (96/603) of such subjects’ anti-VZV concentrations were in the lowest positive concentration group (110-299 mIU/mL). Seropositivity in permanent residents of 1-9 yr old with verified vaccination was merely 61.8 %. Various age groups (1-3 yr, 4-6 yr, and 7-9 yr) all showed seropositivity that gradually decreased with increasing of the interval between vaccination and blood sampling.

**Conclusion:**

Mass varicella vaccination significantly improved the immunity of younger Beijing residents. However, vaccine-induced anti-VZV antibody soon became weak in children with high coverage (approximately 80 %) after vaccination for several years which is significantly higher than reported in pre-licensure studies. A government-funded 2-dose immunization program with mandatory vaccination schedule for Beijing residents may need consideration in the near future.

## Background

Varicella (chickenpox) is a highly contagious disease caused by infection with the primary varicella zoster virus (VZV). Before implementation of the varicella vaccination program in 1995, there were approximately four million varicella cases per year [1] in the United States. Now, varicella vaccines are available globally, some countries have introduced them into the routine immunization program for children, which has succeeded in reducing the morbidity and mortality [2–4]. Numerous post-licensure studies, mostly from field outbreak surveys, have showed the field efficacy and insufficiency of one-dose varicella vaccine in outbreaks control [5]. Based on data obtained from the above-mentioned surveillance and field survey evidence, several countries such as the United States have begun to recommend a second dose of varicella vaccine. However, the post-licensure serological evidences after mass immunization are relatively much less reported [6].

Varicella was once considered a benign and common childhood illness in the People’s Republic of China (PRC) as well as in other countries; consequently, epidemiological and serological data pre-licensure was rarely available. The first varicella vaccine (Varilrix) was initially introduced in PRC by GlaxoSmithKline Biologicals (Rixensart, Belgium) in 1998. Vaccination was not widely accepted in PRC until the introduction of two domestic vaccines (Changchun Keygen Biological Products Co., Ltd, Jilin, PRC and Shanghai Institute of Biological Products, Shanghai, PRC, both licensed in 2000). Till date, there are total 4 vaccines licensed from domestic manufacturers in PRC. Varicella vaccine has not been included in the Expanded Program on Immunization at the national level. In the capital city of Beijing, all vaccines for all age groups are administered in public immunization clinics, and there is no mandatory vaccination schedule of varicella vaccine, which means that any child older than one year can be vaccinated at any time. In Beijing, the clincal varicella cases was notifiable since December 2006, with a stable reported incidence in 2007-2010. In 2010, a survey yielded a data that single-dose vaccine coverage was achieved in 80.4 % of children 3-6 yr old [7]. However, outbreaks caused by breakthrough varicella cases occurred frequently in schools and kindergartens with high single-dose vaccine coverage [8]. Thereafter, a cross-sectional serological survey was performed in order to determine the VZV seroprevalence in the one-dose era and the risk factors of VZV infection in the whole population, which would ultimately provide evidence-based references for developing and adjusting the immunization strategy.

## Methods

### Survey design and subjects

The study was approved by Medical Ethics Committee of the Beijing center for disease control (CDC) and launched in June 2012. A multistage stratified random sampling method was designed to recruit subjects. According to geographic stratification, half of the urban, suburban, and rural districts/counties (9/18) were sampled as research sites. Ten communities/villages in each district/county were systematically sampled as the survey spots. At each survey spot, 20 subjects equally divided between permanent residents and migrants as well as divided between ten age groups (0, 1-4, 5-9, 10-14, 15-19, 20-24, 25-29, 30-34, 35-39, ≥40 yr) were recruited. The total sample size was expected to reach 2 000 people, with approximately 200 subjects of each age group, and 50% each of permanent residents and migrants. After the participants signed informed consent by themselves or by parents/guardians in the case of children, trained doctors used a standardized questionnaire along with a face-to-face interview to collect the following information: sex, age, occupations, household registration, recalled varicella history, and self-reported vaccine administration history, which were also completed by the parents/guardians in the case of children. In order to obtain reliable proof, we verified the vaccination record cards of permanent resident children 1-9 yr old.

To compare with previous serological data, we used blood samples that were collected in another seroepidemiological survey of epidemic cerebrospinal meningitis in Beijing residents in 2008 to assess the differences and trends of anti-VZV seroprevalence in the past 5 years. This study appointed six districts/counties as research sites, including one urban, two suburban, and three rural districts/counties. Each research site had recruited approximately 220 to 240 subjects. Most of the recruited subjects were children and adolescents. Unfortunately, this study did not collect sufficient information of the subjects, except for age, sex, and region, as well as vaccination history.

### Laboratory testing

All samples were stored at 7 °C and transported to the Beijing CDC within 24 hr, then centrifuged and frozen at −20 °C. Samples were tested for IgG-class antibodies to VZV with quantitative enzyme immunoassays according to manufacturer instructions. The serological test is a microplate enzyme-linked immunosorbent assay system that uses purified antigen (cell lysate of a human fibroblast cell line, VZV wild strain) to detect VZV IgG (EUROIMMUN Anti-Varicella-Zoster-Virus IgG-ELISA; Medizinische Labordiagnostika AG, Lübeck, Germany). Positive results (≥110 mIU/mL) were considered immune, negative (<80 mIU/mL) and equivocal results (between 80 and 110 mIU/mL) were considered as not immune.

### Statistical analyses

The database was created with Microsoft Excel (version 2007, Microsoft Corporation, USA). All statistical analyses were performed with SPSS, version 17 (SPSS Inc., Chicago, Illinois, USA). Frequencies were calculated for categorical variables. We used Kruskal-Wallis or Mantel-Haenszel Chi-square tests to evaluate the distribution of continuous or categorical variables and a P-value of <0.05 was considered as significant. The overall seroprevalence was age- and sex-adjusted according to an official 2010 population composition of Beijing residents, which was obtained from the National population Census that is launched every five years.

## Results

### Demographic characteristics of the subjects

A total of 2 144 subjects were recruited in the 2012 survey. Male and female subjects comprised approximately 50 % each; a similar proportion was ensured regarding permanent residents and migrants. People from urban, suburban, and rural areas accounted for 20.9 %, 21.5 %, and 57.6 % of the subjects, respectively. Seven age groups showed successful recruitment of more than 200 subjects. The other three age groups were unable to do so (20-24 yr: 184 subjects; 25-29 yr: 185 subjects; 35-39 yr: 196 subjects). A total of 7.4 % of the overall subjects acknowledged to suffering from varicella once; this was 0 %, 0 %, 4.9 %, and 7.3 % in the age groups <1 yr, 1-4 yr, 5-9 yr, and 10-14 yr, respectively.

In the 2008 survey, 1 421 subjects were recruited. The proportion of male and female subjects was 44.5 % and 55.5 %, respectively. Subjects from urban, suburban, and rural areas accounted for 17.0 %, 33.5 %, and 49.5 % subjects, respectively. Most of the subjects (1 056/1 421) were below 20 yr old.

There was significant difference of geographic regions (*P* < 0.001), sexes (*P* = 0.007), and age group distribution (*P* < 0.001) between the subjects of the 2012 and 2008 studies. The data regarding household registration and occupation distribution was not available for comparison due to this data not being collected in the 2008 survey (Table 1).

**Table 1:** Demographic characteristics of subjects in serological surveys of Beijing Residents in 2012 and 2008, PRC

	Year 2012 (*N* = 2144)	Year 2008 (*N* = 1421)	*P* value
	n (%)	n (%)	
Geographic region^a^			<0.001
Urban	440 (20.5)	241 (17.0)	
Suburb	464 (21.6)	476 (33.5)	
Rural	1240 (57.8)	704 (49.5)	
Gender			0.007
Male	1054 (49.2)	632 (44.5)	
Female	1090 (50.8)	789 (55.5)	
Age			<0.001
<1 year	196 (9.1)	171 (12.0)	
1-4 years	208 (9.7)	350 (24.6)	
5-9 years	225 (10.5)	222 (15.6)	
10-14 years	219 (10.2)	132 (9.3)	
15-19 years	221 (10.3)	181 (12.7)	
20-29 years	430 (20.1)	119 (8.4)	
≥30 years	645 (30.1)	246 (17.3)	
Residency			
Permanent residents	1086 (50.7)	NA	
Migrants	1058 (49.3)	NA	
Recalled Varicella History			
Acknowledge	158 (7.4)	NA	
Deny	1986 (92.6)	NA	

^a^Geographic regions within Beijing were defined as rural, suburb and urban according to the level of urbanization in each district/county

### Seroprevalence comparison: 2012 versus 2008

Seroprevalence was seen in 72.9 % (1 572/2 144) subjects aged 0-74 yr; the age- and sex- adjusted overall anti-VZV seropositivity was 84.5 % as seen in the 2012 survey. Subjects aged ≥30 yr recorded the highest seroprevalence rate (93.7 %, 1212/1293), while the lowest rates were recorded for subjects aged <1 yr (16.9 %, 13/77). In the 2008 survey, 54.8 % (778/1 421) subjects aged 0-76 yr were seropositive; however, the age- and sex-adjusted overall anti-VZV seropositivity was 83.5 %. There was no statistically significant difference in the adjusted overall anti-VZV seropositivity of Beijing residents in 2008 and that in 2012 (*P* = 0.243).

Two age groups showed an increase in sex-adjusted seropositivity (< 1 yr old: from 6.3 % in 2008 to 16.9 % in 2012, *P* = 0.019; 1-4 yr old: from 27.6 % in 2008 to 57.2 % in 2012, P = 0.006). No significant differences were found in other age groups (5-9, 10-14, 15-19, 20-29, and ≥ 30 yr old). (Table 2)

**Table 2:** Anti-VZV Seroprevalence of Beijing residents in 2012 and 2008, PRC

	VZV+, n (%)		Adjusted VZV+, n (%)^*a*^		*P* value^**^
	Year 2012 (*N* = 2144)	Year 2008 (*N* = 1421)	Year 2012 (*N* = 2144)	Year 2008 (*N* = 1421)	
Overall	1559 (72.7)	778 (54.8)	1822(84.5)	1187(83.5)	0.243
Geographic region					
Urban	315 (71.6)	124 (51.5)	NA	NA	
Suburb	357 (76.9)	277 (58.2)	NA	NA	
Rural	887 (71.5)	377 (53.6)	NA	NA	
Gender					
Male	734 (69.6)	297 (47.0)	922 (87.5)	598 (94.6)	<0.001
Female	825 (75.7)	481 (61.0)	885 (81.2)	589 (74.7)	0.001
Age					
<1 year	33 (16.8)	12 (7.0)	13 (16.9)	3 (6.3)	0.019
1-4 years	119 (57.2)	98 (28.0)	31 (57.2)	10 (27.6)	0.006
5-9 years	114 (50.7)	93 (41.9)	27 (50.4)	15 (42.0)	0.438
10-14 years	147 (67.1)	84 (63.6)	79 (67.1)	50 (64.0)	0.651
15-19 years	169 (76.5)	152 (84.0)	221 (76.4)	161 (84.0)	0.058
20-29years	372 (86.5)	106 (89.1)	224 (86.4)	150 (87.5)	0.721
≥30 years	605 (93.8)	233 (94.7)	1212 (93.7)	798 (93.1)	0.593
Household Registration					
Permanent residents	804 (74.3)	NA	NA	NA	NA
Migrants	762 (71.5)	NA	NA	NA	NA

^*a*^Overall VZV+ n and positivity were age- and sex- adjusted. Male and female VZV+ n and positivity were age-adjusted. Age-specific VZV+ n and positivity were sex- adjusted. Adjustments were according to a standard 2010 China population coming from National population Census. ^**^Comparison of the unadjusted and adjuested VZV+ positivity respectively, year 2012 vs. year 2008

### Self-reported varicella vaccine administration history

A total of 26.3 % (563/2 144) subjects reported having received one-dose vaccine, which was 71.6 % (149/208), 80.9 % (182/225), 82.2 % (180/219), and 29.0 % (64/221) in the age groups 1-4 yr, 5-9 yr, 10-14 yr, and 15-19 yr, respectively. Most subjects older than 20 yr reported their vaccination history as unknown. Permanent residents’ vaccine coverage (30.8 %, 335/1086) was slightly higher than that of the migrants (25.5 %, 270/1058). (Table 3)

**Table 3:** Varicella Vaccine administration history of subjects in serological surveys of Beijing Residents in 2012, PRC

	Self-reported vaccination history (*N* = 2144)		
	0-dose, n (%)	1-dose, n (%)	Unknown, n (%)
Age			
<1 year	196 (100.0)	0 (0)	0 (0)
1-4 years	59 (28.4)	149 (71.6)	0 (0)
5-9 years	43 (19.1)	182 (80.9)	0 (0)
10-14 years	32 (14.6)	180 (82.2)	7 (3.2)
15-19 years	70 (31.7)	64 (29.0)	87 (39.3)
20-29 years	75 (17.4)	22 (5.2)	333 (77.4)
≥30 years	66 (10.2)	6 (0.8)	573 (88.8)
Residency			
Permanent residents	270 (24.9)	335 (30.8)	481 (44.3)
Migrants	271 (25.6)	270 (25.5)	519 (49.1)
Total	541 (25.2)	563 (26.3)	1000 (46.5)

### Anti-VZV concentration in the 2012 survey

The mean anti-VZV concentration was 1574.9 (95 % CI 1503.9- 1645.9) mIU/mL. In the age-specific analysis, subjects aged <1 yr were the lowest, with a mean anti-VZV concentration of 103.0 (95 % CI 47.4- 158.5) mIU/mL. The mean anti-VZV concentration increased steadily with increasing age, reaching the highest in subjects aged ≥30 yr, with a mean of 2141.2 (95 % CI 47.4- 158.5) mIU/mL (*P* < 0.001).

There were statistically significant differences in the anti-VZV concentrations between vaccination status (*P* < 0.001) and age groups (*P* < 0.001), but no difference was seen between sexes (*P* = 0.221). What is noteworthy is that 36 % (216/603) of subjects who had received the anti-VZV concentrations of the one-dose vaccine are negative (<110 mIU/mL); additionally 15.9 % (96/603) of such subjects’ anti-VZV concentrations belonged to the lowest positive concentration group (110-299 mIU/mL).

**Fig. 1:**
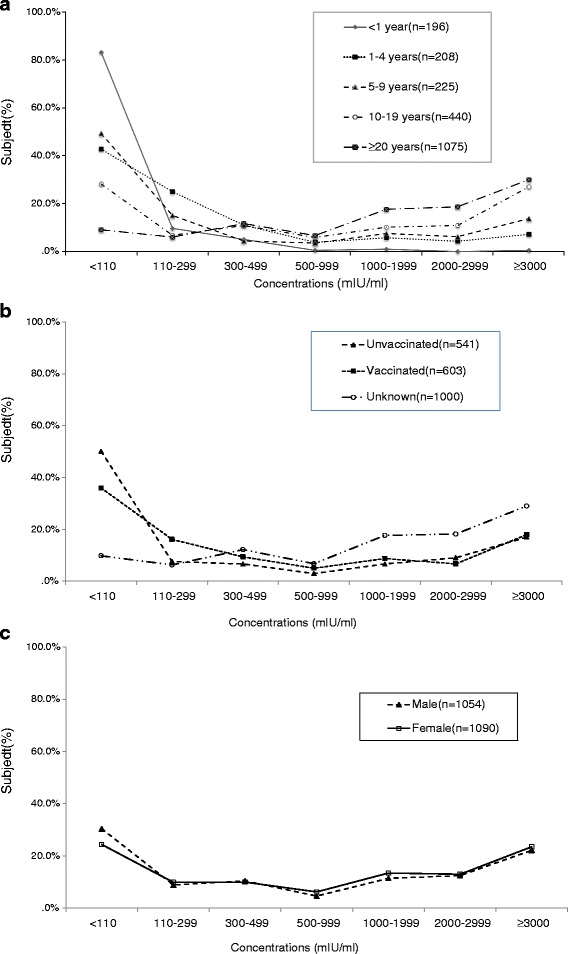
Distribution curves of anti-VZV concentration of Beijing residents in 2012 by **a** age group, **b** gender and **c** vaccine vaccination (*n* = 2157). There were statistically significant differences in anti-VZV concentrations between age groups (*P* < 0.001) and vaccination status (*P* < 0.001), but no difference between gender (*P* = 0.221), by *x* ^2^ test

Reversal phenomenon was also found in the age-specific composition of different anti-VZV concentration groups. The proportion of subjects aged 5-9 yr (49.3 %, 111/225) was higher than that of subjects aged 1-4 yr (42.8 %, 89/208) in the negative concentration group (<110 mIU/mL); however, this (15.1 %, 34/225) immediately changed and was lower in subjects aged 1-4 yr (25.0 %, 52/208) in the lowest positive concentration group (110-299 mIU/mL) (Fig. 1).

### Seroprevalence of permanent residents aged 1-9 years based on verified vaccination records

**Fig. 2:**
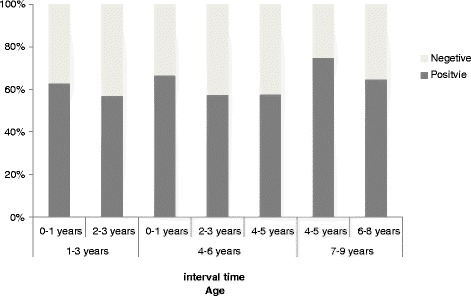
Seroprevalence of 1-9 years permanent Beijing residents in 2012 who denied varicella history and had received one dose vaccine based on verified vaccination records. Distribution of different age groups by different between vaccination and blood sampling interval (*n* = 165)

Based on verified vaccination records, 19.5 % (44/226) permanent residents who were 1-9 yr old in 2012 had not received vaccine and another 1.3 % (3/226) subjects acknowledged suffering from varicella. Thus, seroprevalence in a total of 165 subjects was analyzed based on different age groups and different intervals between vaccination and blood sampling. The seropositivity of the 1-3 yr, 4-6 yr, and the 7-9 yr groups were 61.5 % (32/52), 58.5 % (38/65), and 66.7 % (32/48), respectively, a total of 61.8 % (102/165). All three age groups showed gradual decrease in seropositivity with increasing interval between vaccination and blood sampling (Fig. 2).

## Discussion

To the best of our knowledge, our study is the first to present the serological immunity against varicella as a trend among all age groups of different residents in the single-dose population and voluntary vaccination era in Beijing, PRC. The overall and age-specific anti-VZV seropositivity is slightly higher than seen in another study reported by Shanghai scholars in 2012, where the estimated one-dose vaccine coverage was 69.4 % in children aged 4-6 ys [9]. This incidence is still lower than that of countries that have introduced vaccines in the national routine immunization schedule, such as Korea [10] (89.6 %, 1-79 yr old in 2013) and the US [11] (93.6 % of 6-19 yr old and 98.0 % of 20-49 yr old in 1999-2004) with similar one-dose immunization indications.

Passive surveillance data provided the varicella epidemiological characteristics and vaccine coverage in Beijing [7], which reports a stable annual incidence (range:1.0-1.1/1 000 persons), a significant decrease of incidence in children 1-4 yr old (vaccination age of most children), and a peak incidence in children 5-9 yr old between 2007 and 2010. It also illustrated that estimated single-dose vaccine coverage could reach up to 80.4 % in children 3-6 yr old in 2010.That means for each birth cohort a few years earlier than 2010, vaccination coverage at a younger age (1-2 yr) is low (usually less than 50 %), which then increases with age; a higher vaccination coverage (approximately 80 %) can be achieved at an older age (5-9 yr). Our study found that the adjusted overall anti-VZV seroprevalence in two groups in 2012 showed significant growth compared with 2008 (< 1 yr old: from 6.3 % to 16.9 %; 1-4 yr old: from 27.6 % to 57.2 %). This finding may indicate the success of mass varicella vaccinations in increasing the immunity in younger Beijing residents.

In clinical trials of the single-antigen varicella vaccine that were conducted pre-licensure, seroconversion was assessed by using several vaccines with different amounts of PFUs and laboratory assays with different levels of sensitivity and specificity. These studies usually reported high short-term seroconversion [12, 13] after vaccination as well as long-term persistence of antibody [14], especially in vaccinated children. Relatively few post-licensure studies reported only 76 % seroconversion by using validated sensitive and specific fluorescent antibodies to membrane antigen (FAMA) assay [6]. Our study found that 36 % (216/603) of the subjects who received one-dose varicella vaccine were negative. Especially, the seropositivity of permanent residents 1-9 yr old and older with verified one dose vaccine was merely 61.8 %. Different age groups (1-3 yr, 4-6 yr, and 7-9 yr) all showed gradually decreasing seropositivity with the increasing of the interval between vaccination and blood sampling. Two types of domestic vaccines we focused on had reported good and similar immunogenicity [15] (Changchun VS Shanghai: 92.3 % VS 88.3 %; total 659 children of 2-6 yr old; according to FAMA test). However, another early clinical trial reported that the Shanghai vaccine immunogenicity was 96 % after vaccination, which significantly decreased to 81.4 % two years later [16]. It is speculated that, in addition to the reasons for the primary immunization failure, the rate of antibody decline is faster than seen in pre-licensure studies, which may be due to asymptomatic boosting of vaccine-induced immunity, and the fact that exposure to wild-type VZV is more impossible after extensive application of vaccines and a dramatic decline in incidence.

The World Health Organization recommends 2-dose routine childhood immunization against varicella in countries ensuring a vaccine coverage ≥80 %, as seen in the latest position paper [17]. The ACIP has adopted new recommendations for a routine 2-dose varicella vaccination program for children since 2006 with the second dose given at age 4-6 yr old [18]. The first recommendation for the booster dose of the varicella vaccine for children ≥4 yr old in PRC was published in October 2012 by the Beijing CDC. Additionally, a catch-up vaccination was also recommended for children and adolescents who had previously received the one-dose vaccine [19]. Nevertheless, the varicella vaccine has still not been introduced in the national immunization program until now, the single-dose vaccine coverage through voluntary vaccination strategies has almost reached the maximum limit, and there is still uncertainty regarding booster dose coverage. Given the above challenges, a government-funded 2-dose immunization program with mandatory vaccination schedule for Beijing residents may need consideration in the near future.

Our study has some limitations. The 2012 survey covered the whole population, with adolescents and adults ≥15 yr old accounting for more than 60 %, but most of the recruit subjects were children and adolescents in the historical data from the 2008 survey. The lack of adult sample may therefore affect the comparison of the overall anti-VZV seropositivity of the entire population. Seroprevalence evaluated by using ELISA does have limitations in sensitivity and specificity of the method [20, 21] which may underestimate the seropositivity of the antibody induced by the vaccine. It is, however, not a problem for us to comprehensively trace the entire picture of varicella seroprevalence in Beijing residents from the single-dose vaccine indication era. This can provide us a view into the degree of susceptibility and infection risks in populations of different ages. One more topic worth exploring is the immunization failure occurring in the specific age group that received one-dose vaccine after mass vaccination, which is significantly higher than reported in previous studies.

## Conclusion

In conclusion, our study found that mass single-dose vaccination caused significant higher seropositivity in subjects aged <1 yr and 1-4 yr in 2012 compared with 2008. However, vaccine-induced anti-VZV antibody soon became weak in children with high coverage (approximately 80 %) after vaccination for several years. This serological evidence is mutually validated by data obtained from many field epidemiological evidences, such as outbreaks surveys, which prove that the effectiveness of one-dose varicella vaccine is insufficient. This provides us with another theoretical hypothesis to support and suggest a government-funded vaccination program including catch-up vaccination for children that needs to be considered in the near future in Beijing.

## Abbreviations

Beijing CDC: Beijing municipal center for disease control and prevention
VZV: Varicella zoster virus
anti-VZV: Anti VZV IgG antibodies
PFU: Plaque forming unit
ACIP: The advisory committee on immunization practices

## References

[1] Seward JF, Watson BM, Peterson CL (2002). Mascola L, Pelosi Jw, Zhang JX, et al. Varicella disease after introduction of varicella vaccine in the United States, 1995–2000. JAMA.

[2] Nguyen HQ, Jumaan AO, Seward JF (2005). Decline in mortality due to varicella after implementation of varicella vaccination in the United States. N Engl J Med.

[3] Marin M, Meissner HC, Seward JF (2008). Varicella Prevention in the United States: a review of successes and challenges. Pediatrics.

[4] Streng A, Grote V, Carr D, Hagemann C, Liese JG (2013). Varicella routine vaccination and the effects on varicella epidemiology-results from the Bavarian Varicella Surveinllance Project (BaVariPro), 2006–2011. BMC Infect Dis..

[5] Seward JF, Marin M, Vazuqez M (2008). Varicella vaccine effectiveness in the US vaccination program: a review. J Infect Dis.

[6] Michalik DE, Steinberg SP, Larussa PS, Edwards KM, Wright PF, Arvin AM (2008). Primary vaccine failure after 1 dose of varicella vaccine in healthy children. J Infect Dis..

[7] Lu L, Wang C, Suo L, Li J, Liu W, Pang X, Seward JF (2013). Varicella disease in Beijing in the era of voluntary vaccination, 2007 to2010. Pediatr Infect Dis J.

[8] Lu L, Suo L, Li J, Zhai L, Zheng Q, Pang X, Bialek SR, Wang C (2012). A varicella outbreak in a school with high one-dose vaccination coverage, Beijing, China. Vaccine.

[9] Yang Y, Tang S, Liu J, Li Z, Lu H, Chen W, Wu Q, Guo X, Li C, Sun X, Xu W (2012). Survey of antibody levels to varicella-zoster virus in healthy population in Shanghai. Disease surveillance.

[10] Lee H, Cho HK, Kim KH (2013). Seroepidemiology of varicella-zoster virus in Korea. J Korean Med Sci.

[11] Reynolds MA, Kruszon-Moran D, Jumaan A, Schmid DS, McQuilan GM (2010). Varicella seroprevalence in the U.S.: date from the National Health and Nutrition Examination Survey, 1999-2004. Public Health Rep.

[12] Merck & Co., Inc (1995). VARIVAX [Package insert].

[13] White CJ, Kuter BJ, Hildebrand CS, Isganitis KL, Matthews H, Miller WJ (1991). Varicella vaccine (VARIVAX) in healthy children and adolescents: results from clinical trials, 1987 to 1989. Pediatrics..

[14] Kuter B, Matthews H, Shinefield H, Black S, Dennehy P, Watson B (2004). Ten year follow-up of healthy children who received one or two injections of varicella vaccine. Pediatr Infect Dis J..

[15] Lin Y, Wang J, Chao J, Shen G, Jiang Y, Lu J (2004). Comparison of immunogenicity and safety of two Domestic Freeze-dried Live attenuated varicella vaccine. Chin J Epidemiol.

[16] Sun H, Yuan J, Liu X (2003). The serological immunization effect and cost benefit analysis of one domestic Freeze-dried Live attenuated varicella vaccine. Sh J Prev Med.

[17] Varicella and herpes zoster vaccines: WHO position paper, June 2014.WHO Weekly Epidemiol Rec. 2014; 89:265-288. 24983077

[18] Marin M, Güris D, Chaves SS, Schmid S, Seward JF (2007). Advisory Committee on Immunization Practices, Centers for Disease Control and Prevention (CDC). Prevention of varicella: recommendations of the Advisory Committee on Immunization Practices. MMWR Recomm Rep.

[19] Lu L, Suo L, Li J, Beijing CDC (2013). The technical guidelines of the use of varicella vaccine in Beijing City. Chin J Prev Med.

[20] Sauerbrei A, Wutzler P (2006). Serological detection of varicella-zoster virus-specific immunoglobulin G by an enzyme-linked immunosorbent assay using glycoprotein antigen. J Clin Microbiol.

[21] Sauerbrei A, Schafler A, Hofmann J, Schacke M, Gruhn B, Wutzler P (2012). Evaluation of three commercial varicella-zoster virus IgG enzyme-linked immunosorbent assays in comparison to the fluorescent-antibody-to-membrane-antigen test. Clin Vaccine Immunol.

